# Influence of Different Repair Acrylic Resin and Thermocycling on the Flexural Strength of Denture Base Resin

**DOI:** 10.3390/medicina56020050

**Published:** 2020-01-21

**Authors:** Mohammed AlQahtani, Satheesh B. Haralur

**Affiliations:** 1 Department of Prosthetic Dental Science, College of Dentistry, King Saud University, Riyadh 11454, Saudi Arabia; moqahtani@ksu.edu.sa; 2 Department of Prosthodontics, College of Dentistry, King Khalid University, Abha 62529, Saudi Arabia

**Keywords:** polymethylmethacrylate, denture fracture, denture repair, light cure acrylic, flexural strength, self-cure acrylic

## Abstract

*Background and Objectives:* Fractured acrylic denture base is a common occurrence in clinical practice. The effective denture repair procedure is cost-effective, time conserving, and results in lesser time without denture for the patient. Along with various reinforcements and surface modifications; different acrylic resins are investigated in improving the flexural strength of the fractured site. The aim of this study was to evaluate the flexural strength of a polymethyl methacrylate (PMMA) denture base repaired with heat-polymerized (HPA), auto-polymerized (APA) and light-polymerized acrylic (LPA) resins after thermocycling. *Materials and Methods:* Forty rectangular shaped (50 mm × 25 mm × 3 mm) PMMA specimens were fabricated. Group 1 specimens (*n* = 10) were kept as controls and the remaining 30 samples were sectioned at the center with a repair site dimension of 3 mm. The samples from three groups (*n* = 10) were repaired with HPA, APA, and LPA resins, respectively. The specimens were thermocycled for 5000 cycles and subjected to a three-point flexural test. The maximum load required to fracture the specimens was recorded, and further analyzed with ANOVA and the Games-Howell Post hoc test at the significance level *p* = 0.05. *Results:* The average maximum load and flexural strength of the control group was 173.60 N and 13.02 Mpa and corresponding values for denture repaired with HPA was 87.36 N and 6.55 Mpa. The corresponding values for APA resin and LPA resins were 62.94 N, 57.51 N, and 4.72 Mpa, 4.06 Mpa, respectively. *Conclusions:* The PMMA specimens repaired with HPA resins resulted in a significantly higher load to fracture compared to APA resin and LPA resin.

## 1. Introduction

Conventional complete denture and partial denture are still preferred as a treatment choice to replace missing teeth, for medical and financial reasons [1]. Polymethyl methacrylate (PMMA) is frequently used to fabricate complete or partial denture bases because of its numerous advantages, including low cost, biocompatibility, ease of processing, stability in the oral environment, and satisfactory aesthetics [2]. The cracking and fracturing of denture bases is still an unresolved clinical complication in dental practice. It is the primary cause of the failure of the removable prosthesis [3]. Denture base fracture is primarily attributed to poor mechanical properties like low impact strength and reduced fatigue resistance [4]. The prosthesis may fracture due to impact force during accidental fall or fatigue failure in the course of service. Fatigue failure is caused by the repeated denture flexure from the occlusal force. The progressive resorption of the supporting bone foundation leads to denture instability and movement during mastication. Constant exposure to innumerable stress cycles with improper denture support results in stress accumulation and fatigue failure [5]. The maxillary denture is mostly fractured in the midline running through labial frenulum due to tensile stress from the masticatory forces [6]. Successful prosthetic rehabilitation depends on the diligent balance of static and dynamic forces generated from soft and hard tissues around the denture border. Managing the forces in oral physiology by careful consideration of teeth volume, angulation, volume, neutral zone, and residual ridge morphology is vital for long-term success of denture prosthesis. [7,8]

Eklund [9] and Caplan D [10] reported a higher risk of edentulism among the community with lower levels of education and income status. The choice of dental prosthesis is influenced by multiple factors such as aesthetic expectations, cost, and patient acceptability. A higher preference for the removable partial denture is observed in lower socioeconomic groups due to affordability and accessibility. Though removable partial dentures are fabricated with cast metal, and all-acrylic materials; the use of all-acrylic removable partial denture prosthesis fabrication is frequently reported in developing countries [11]. Consequently, developing cost-effective denture repair techniques and materials is imperative considering the section of society utilizing the all-acrylic denture. The objectives of efficient denture base repair must have adequate strength, be inexpensive, be an easy procedure, have dimensional accuracy, and allow color matching with bulk material. The denture repair procedure endeavor is to redevelop the original strength of the denture to prevent further fracture [12]. The heat-polymerizing acrylic (HPA) resin, auto-polymerizing acrylic (APA) resins, and light-polymerizing acrylic (LPA) resins are frequently employed for the denture fracture repair [13]. HPA resins are preferred due to their higher mechanical properties [14]. However, the repair procedure requires the custom-made gypsum mold and is time-consuming. Denture fracture repair with APA resins is economical and less time consuming; the common shortcomings are poor fracture strength, residual monomer content, and dimensional inaccuracies. Few researchers prefer using LPA resin for denture repair due to a higher modulus of elasticity and dimensional accuracy [15].

The strength of denture repairs is primarily determined by the factors affecting adhesion and mechanical properties of repair resins. Researchers attempted to improve fracture resistance by incorporating various reinforcing agents like glass fiber [16], carbon fiber [17], metal mesh [18], and polyethylene fibers [19]. Alternatively, surface treatment of the fracture site with acetone [20] and chloroform [21] was recommended by a few investigators. Previous researchers have also suggested different fracture edge profile modifications [22] and roughened internal surface [23] to enhance the surface area. Additionally, the denture base material and repair acrylic resin combination also affect the strength of denture repair. The denture repair resin is selected on multiple criteria like working time, mechanical properties, and dimensional stability during and after repair [24].

The denture fracture is also predisposed due to the chemical and mechanical factors inside the oral cavity. The denture is constantly immersed within the saliva, and other consumed beverages. The temperature variation and pH of these liquids contribute to the deterioration of the mechanical properties of the denture base [25]. The oral fluids are absorbed into the denture polymer and act as a plasticizer and weaken the material [26]. Although the previous studies have explored the flexural strength of acrylic resins, the effect of thermocycling on the flexural strength of repaired resins are not comprehensively investigated. The comparative performance of HPA, APA, and LPA resins when used as denture repair resins also needs further investigation.

Hence, the purpose of this study was to evaluate the maximal flexural strength of a polymethyl methacrylate denture base repaired with heat-polymerized, auto-polymerized, and light-polymerized acrylic resins after thermocycling. The hypothesis of the study was that no difference existed in flexural resistance between the denture base repaired with various denture repair acrylic resins after thermocycling.

## 2. Materials and Methods

### 2.1. Preparation of the Samples

A total of forty rectangular-shaped specimens from heat-polymerized polymethyl methacrylate denture base resin (Major Base 20, Major Prodotti Dentari S.p.A.Einaudi Moncalieri Italy), with the dimensions of 50 mm × 25 mm × 3 mm, were fabricated. Following the manufacturer’s instructions, the PMMA acrylic resin was mixed with a powder:liquid ratio of 100 g to 43 mL. The resin was packed at the dough stage into a stainless steel mold. The resin was heat-polymerized by immersing it in temperature-regulated acrylizer (Acrydig 12, Manfredi Srl, San Secondo di Pinerolo (TO), Italy). The temperature of the water was kept at 740 °C for 90 min, followed by 1000 °C for 30 min. Post heat-curing, the denture flasks were allowed to cool down to room temperature. The resin specimens were finished by 600-grit silicone carbide paper under water coolant. The finished specimens were stored in water for 48 h before testing for water saturation. The prepared samples were randomly divided into four groups (*n* = 10). Based on previously published studies [27,28], effect size (d) 1.6, α at 0.05, and 1–β (power) at 0.9 the sample size was determined to be 10 per sub-group, resulting in a total of 40 specimens per group. The sample size was calculated with the G*Power software (version 3.1; University of Dusseldorf) [29]. The fractured site was repaired with heat-polymerized polymethyl methacrylate (HPA) resin for Group 2, auto-polymerized polymethyl methacrylate (APA) resins for Group 3, and light-polymerized urethane methacrylate (LPA) resin for Group 4. Group 1 samples were used as a control group with no fracture and repair sites.

**Figure 1:**
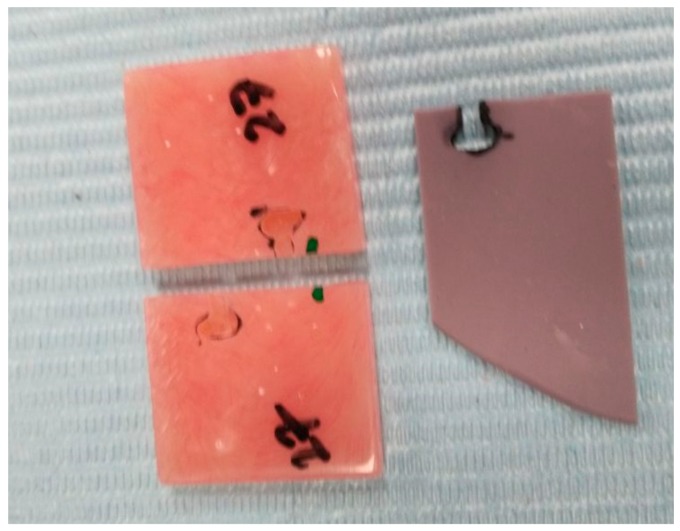
Putty mold to standardize the dovetail dimension on acrylic samples.

Subsequently, each sample was sectioned in the middle into two halves separated by a 4 mm × 25 mm × 3 mm space; using with a thin diamond disk under water coolant. The fragmented specimen’s edge was shaped into a standard round contour. The repair site edges from both segments were pretreated with 50 µm alumina oxide air abrasion under 0.5 Mpa pressure. One dovetail in standardized shape and size was created to each side of the repairing area not apposing to each other (Figure 1).

### 2.2. Repair Procedures

The die stone mold was constructed for repairing the fractured site for Group 2 samples. Following the manufacturer’s instructions, The HPA resin was mixed, flasked, and placed under compression (1250 kg) for 30 min at room temperature. Subsequently, it was heat-cured in a short-cycle water bath for 60 min at 100 °C degrees [30].

**Figure 2:**
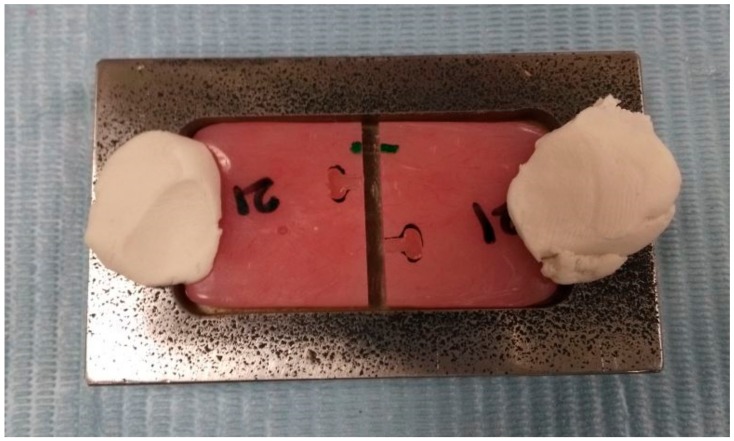
Stainless steel mold used for repairing the fractured acrylic resin samples.

The paired halves of bulk PMMA acrylic samples from Group 3 were placed back in a stainless steel metal mold (Figure 2). The standardized repair space of 3 mm was maintained between segments, the auto-polymerizing acrylic resin (Resine, BMS Dental Srl, Capnnoli, PISA, Italy) was mixed and added in the free-flowing state into the repair site following the manufacturer’s instructions. The flask was closed under pressure (bench press) and polymerized under two bar pressure for 2 h. Post-polymerization, the repair site was finished with 600-grit silicone carbide paper and stored in water for 1 week at a 37 °C temperature.

Group 3 PMMA resin samples were repaired with LPA resin (Eclipse, DENTSPLY International, Inc. Avenue York, PA, USA). The repair site edges were cleaned with isopropyl alcohol and allowed to dry. Baseplate Resin heated with Wax Pencil Pro and flow into the repair site one side to the other to avoid air entrapment. The repair location was filled with the resin and was covered with air carrier coating and cured in a light cure unit (Enterra, DENTSPLY International, Inc. Avenue York, PA, USA) using the repair cure cycle. Post-curing, the repair site was washed with water and finished with 600-grit silicone carbide paper.

### 2.3. Three-Point Flexural Test

Repaired PMMA samples were subjected to thermocycling process in water between 5 °C and 55 °C for 5000 (1100; SD Mechatronik) cycles, with the dwell time of 30 s. The flexural test was conducted using a universal testing machine (Instron Corporation, Norwood, MA, USA) at the crosshead speed of 5 mm/min. The load was applied at the center of the repair site and the maximum load required to fracture the samples was recorded.

### 2.4. Statistical Analysis

The obtained data were evaluated with ANOVA and Games-Howell Post hoc test using SPSS 19 (IBM Corporation, Armonk, NY, USA) at the significance level *p* = 0.05.

## 3. Results

The mean maximum load and flexural strength of each group are summarized in Table 1. The control group, with no repair site, expectedly recorded the highest maximum load at 173.60 N, and flexural strength of 13.023 Mpa. Among the resins utilized for repair, the HPA resins performed better compared to other resins. It showed the maximum load of 87.36 N and a flexural strength of 6.55 Mpa. The APA resins and LPA resins exhibited a maximum load of 62.94 N and 57.51N, respectively. Flexural strength performance of LPA resin was poor; at 4.06 Mpa

A one-way ANOVA between the groups was performed to compare the impact of using different resins to repair PMMA denture base resins (Table 2). The mean of the maximum load of the four groups was unequal according to a one-way ANOVA; F(3, 36) = 309.48, *p* = 0.00. The outcome variable was found to be significantly different with the Welsh test; F(3, 18) = 208.82, *p* < 0.001. The mean of flexural strength between the groups was equally found to be significantly different; F(3, 36) = 343.74, *p* = 0.00. The outcome variables for flexural strength were also found to be significantly different with the Welsh test; F(3, 17) = 207.18, *p* < 0.001.

Table 3 demonstrates the results of the Games–Howell Post hoc pairwise comparison. The result indicated the statistically significant difference between all the groups both in maximum load and flexural strength.

## 4. Discussion

The fracture of PMMA denture base resin remains a persistent clinical problem in prosthodontics. The denture is repaired as an interim or on a few occasions as a permanent solution [31]. The repair methods, apart from being easy and economical, should ensure that the repair possess adequate strength to resist the fracturing during function [15]. This study assessed the comparative flexural strength and the maximum load of the PMMA denture base repaired with HPA, APA, and LPA resins after thermocycling. The repair site dimension was kept uniformly at 3 mm to reduce the bulk of repair material and consequently minimize the polymerization shrinkage [32].

Based on the results of the present study, the null hypothesis of no difference in denture repair site strength between various acrylic resins after thermocycling was rejected. The strength of the denture repair site depends on successful adhesion between repair material and denture base resin [33]. As a consequence, the majority of failures are adhesive in nature [34]. Repairing the fractured PMMA denture base with HPA resin showed higher fracture resistance and flexural strength compared to other acrylic resins. The structural similarity of repair and bulk acrylic resin material possibly could have aided in better chemical bonding and adhesion. Initial low consistency resin mix, along with the presence of the monomer, would dissolve the PMMA fractured edges and form the durable secondary semi-interpenetrating polymer networks [35,36]. Additional exposure to heat during repair is also expected to facilitate the further polymerization of bulk acrylic resin.

Though HPA repair strengths are encouraging, it is only occasionally utilized because of multiple unfavorable factors, such as; additional laboratory costs due to requiring the fabrication of a split gypsum mold; probabilities of heat-induced deformation; prolonged polymerizing processes; and lack of denture for the patient during repairing procedure. Auto-polymerizing PPMA is preferred for repairing the fractured denture base due to its easy, quick, and economical laboratory procedure. The findings from previous studies [37,38,39] are contradictory regarding the flexural strength of auto-polymerized resin compared to heat-polymerized resin. Few authors report the inferior strength of auto polymerized resin, while Agarwal M et al. [40] and Rached [41] reported a similar strength with heat-polymerized resin. Frequently, the repaired denture base is found to be fractured again at the repair site. Researchers are of the opinion that this could be due to lower transverse strength of auto-polymerizing acrylic resin. The lesser degree of polymerization from chemical polymerization initiators are attributed to its poor transverse strength of APA resins [34]. Earlier research reported a denture base repaired with APA resins to have approximately 60–65% of the strength of unrepaired PMMA acrylic resin [35,38], while a denture repaired with heat-cured acrylic resin demonstrated 75–85% of the strength of the original un-fractured PMMA bulk material [42]. Although, our study showed a similar repair strength between the various acrylic resins. The heat-polymerized acrylic resin performed at 51% strength of original bulk material. The difference could be due to the subjecting of the repaired resins to thermocycling procedures in our study. The results are in agreement with earlier research of reduced flexural strength after thermocycling [43,44]. The reduction of fracture strength after thermocycling could be ascribed to water absorption, thermal stress, and presence of porous structures [45].

LPA resins are preferred by few clinicians to overcome the limitations of both heat- and auto- polymerized acrylic resins. LPA resins provide multiple advantages like lesser chemical irritation due to lower residual monomer content, and good color stability [46,47]. On the contrary, it exhibits higher water absorption, less impact strength, and poor adhesion to denture teeth [48]. In our study results, LPA resins showed the least fracture resistance and flexure strength compared to other acrylic resins. Dar Oden et al. [49] reported the higher transverse strength from APA resin compared to LPA resin as a repair material for PMMA denture base. Similar findings were reported by Polyzois et al. [50], Andreopoulos et al. [51], and Jagger et al. [52]. Meanwhile, Lewinstein et al. [15] described an insignificant difference between APA and LPA resin bond strength and a heat-cured PMMA denture base. The LPA resins are routinely mixed and adapted over the repair site manually. Additionally, it is polymerized without the pressure. Hence, the high probability of incorporation of internal voids and defects result in a compromised mechanical performance [36]. LPA resin dough may not infiltrate the PMMA polymer network in the same way as other repair resins with more residual monomers and lesser viscosity resins. Improved bond strength of APA and HPA resins to the bulk PMMA resin could be due to exposure of its fractured edges to methyl methacrylate monomer. Previous research indicated that the surface treatment with methyl methacrylate leads to the softening of fractured edges [24], formation of pits, and promotion of superficial crack propagation [53]. These surface irregularities facilitate the diffusion of repair material and enhance adhesion [54].

This in vitro study has limitations in simulating clinical situations, in which denture design is different from tested samples. The occlusal load is repetitive in nature; hence, the denture fracture is a result of fatigue failure. The denture made during clinical service immersed in saliva and various beverages. Hence, further studies are required to evaluate the effect of saliva and the resilient nature of denture-supporting tissues.

## 5. Conclusions

Within the limitations of this in-vitro study, the following conclusions were drawn.

1. The Heat cured PMMA denture base repair with heat polymerized acrylic resin provided the highest fracture resistance and flexural strength.
2. Light polymerized acrylic resin used for repairing PMMA denture base performed inferior to heat and auto polymerized acrylic resins both in maximum load and flexural strength.
3. Auto-polymerized repair acrylic resins showed a moderately higher flexural strength than light polymerized acrylic resin but showed significantly lesser performance than the heat cure resins.
4. Though heat cure repair acrylic resins, recorded the maximum flexural strength, the denture repair procedures are time-consuming, higher cost and require dental laboratory support. Hence, further researches to simplify the repair process is required.

## Figures and Tables

**Table 1:** Descriptive statistics of the maximum Load (N) and flexure strength (Mpa) recorded in the different groups.

Group	N	Maximum Load	Flexure Strength
		Mean (SD)	Mean (SD)
Control	10	173.60 (18.48)	13.02 (1.29)
Heat PMMA	10	87.36 (4.82)	6.55 (0.45)
APMMA	10	62.94 (1.83)	4.72 (0.13)
UDMA	10	57.51 (2.23)	4.06 (0.21)

**Table 2:** One-Way Analysis of Variance of maximum load and flexural strength between the groups.

Test	Source	*df*	*SS*	*MS*	*F*	*p*
Maximum load	Between Groups	3	86,700.453	28,900.15	309.48	0.000 *
	Within Groups	36	3361.689	93.38		
	Total	39	90,062.141			
Flexural strength	Between Groups	3	501.932	167.31	343.74	0.000 *
	Within Groups	36	17.522	0.48		
	Total	39	519.454			

* The mean difference is significant at the 0.05 level.

**Table 3:** Games–Howell Post hoc pairwise comparison for maximum load and flexural strength between the groups.

Test	Group	Control	HPMMA	APMMA	UDMA
Maximum load	Control	-	0.000 *	0.000 *	0.000 *
	HPMMA	0.000 *	-	0.000 *	0.000 *
	APMMA	0.000 *	0.000 *	-	0.000 *
	UDMA	0.000 *	0.000 *	0.000 *	-
Flexural strength	Control	-	0.000 *	0.000 *	0.000 *
	HPMMA	0.000 *	-	0.000 *	0.000 *
	APMMA	0.000 *	0.000 *	-	0.000 *
	UDMA	0.000 *	0.000 *	0.000 *	-

* The mean difference is significant at the 0.05 level.
